# Surface Permeability of Membrane and Catalytic Performance Based on Redox-Responsive of Hybrid Hollow Polymeric Microcapsules

**DOI:** 10.3390/molecules26030633

**Published:** 2021-01-26

**Authors:** Guangyu Wu, Jingyi Wang, Qi Liu, Ran Lu, Yuhan Wei, Feng Cheng, Jiangang Han, Weinan Xing, Yudong Huang

**Affiliations:** 1 Co-Innovation Center for the Sustainable Forestry in Southern China, College of Biology and the Environment, Nanjing Forestry University, Nanjing 210037, China; wjy15850792685@163.com (J.W.); 15380741292@163.com (Q.L.); luranNJLR@126.com (R.L.); weiyuhan201909@163.com (Y.W.); 2 Key Laboratory of Functional Polymer Materials, Ministry of Education, Nankai University, Tianjin 300071, China; 3 State Key Lab of Fine Chemicals, Dalian University of Technology, Dalian 116024, China; 4 Jiangsu Provincial Key Laboratory of Palygorskite Science and Applied Technology, Huaiyin Institute of Technology, Huai’an 223003, China; 5 National Positioning Observation Station of Hung-tse Lake Wetland Ecosystem in Jiangsu Province, Hongze 223100, China; 6 MIIT Key Laboratory of Critical Materials Technology for New Energy Conversion and Storage, State Key Laboratory of Urban Water Resource and Environment, School of Chemistry and Chemical Engineering, Harbin Institute of Technology, Harbin 150001, China; chengfeng9004@126.com (F.C.); happyliwe@163.com (Y.H.)

**Keywords:** hybrid hollow polymeric microcapsules, membrane, redox-responsive, self-assembly, catalytic performance

## Abstract

“Smart” polymeric microcapsules with excellent permeability of membranes have drawn considerable attention in scientific and industrial research such as drug delivery carriers, microreactors, and artificial organelles. In this work, hybrid hollow polymeric microcapsules (HPs) containing redox-active gold-sulfide bond were prepared with bovine serum albumin, inorganic metal cluster (AuNCs), and poly(*N*-isopropylacrylamide) conjugates by using Pickering emulsion method. HPs were transferred from water-in-oil to water-in-water by adding PEGbis(*N*-succinimidylsuccinate). To achieve redox-responsive membrane, the Au-S bond units incorporated into the microcapsules’ membranes, allowed us to explore the effects of a new stimuli, that is, the redox Au-S bond breaking on the microcapsules’ membranes. The permeability of these hybrid hollow polymeric microcapsules could be sensitively tuned via adding environment-friendly hydrogen peroxide (H_2_O_2_), resulting from a fast fracture of Au-S bond. Meanwhile, AuNCs and conjugates could depart from the microcapsules, and enhance the permeability of the membrane. Based on the excellent permeability of the membrane, phosphatase was encapsuled into HPs and p-nitrophenyl phosphate as a substrate. After adding 1 × 10^−2^ and 1 × 10^−4^ M H_2_O_2_, the catalytic efficiency was nearly 4.06 and 2.22 times higher than that of HPs in the absence of H_2_O_2_, respectively. Hence, the unique redox-responsive HPs have potential applications in biocatalytic reaction, drug delivery, and materials as well as in bioscience.

## 1. Introduction

In recent years, “smart” hollow polymeric microcapsules, with their special structures, have attracted great interest in many kinds of research fields and from several groups of scientists all over the world [1,2,3,4]. The aqueous core surrounded by a membrane integrated chemical ensembles, showing great potential applications in many fields such as biomedicine, catalysis, biosensing, and synthetic protocellular systems. Moreover, these functions are related to the exchange of substances between the inside and outside part. Hest and co-authors used poly (styrene boronic acid) as building blocks, controlling the permeability of polymersome by pH. The designed microcapsules could be used as a nanoreactor. Recently, Huang and co-authors showed that the permeability of microcapsules could be modulated by temperature and redox. Due to the combination of different applications with hollow microcompartment, a way to control the membrane permeable based on environment response is a key concern and should be designed first. Scientists paid more attention to making hollow microcompartments undergo a chemical or physical change in response to environmental stimuli such as pH, temperature, redox species, light, magnetic, and electric fields [5,6,7,8,9]. In general, these chemical or physical changes will lead to the self-assembly of porous membranes, resulting in the breakage or re-formation of channel pores at the edges of the membranes. Among these multiple responses, the use of environment-friendly non-toxic redox response is the most significant.

Herein, a type of hybrid hollow polymeric microcapsules (HPs) was self-assembled with bovine serum albumin (BSA), inorganic metal cluster (AuNCs), and poly(*N*-isopropylacrylamide) conjugates linked via a gold-sulfide bond by using Pickering emulsion technique. Given the redox species sensitive gold-sulfide bond, the constructed HPs were clearly endowed a frequent behavior with a huge modulation of the permeability of membrane by adding environment-friendly hydrogen peroxide (H_2_O_2_). Moreover, the phosphatase (ALP, encapsulated inside HPs) showed a high catalytic efficiency by selected 4-nitrophenyl phosphate (pNPP) as substrate. To the best our knowledge, this is the first time that the permeability of membrane of hybrid redox-responsive hollow polymeric microcapsules is reported.

## 2. Materials and Methods 

### 2.1. Materials

1-Propanethiol (Aladdin, Shanghai, China, 99%), carbon disulfide (Sigma, Metairie, LA, USA, 99%), potassium ferricyanide (K_3_Fe(CN)_6_, Aladdin, Shanghai, China, 99%), 4,4′-azobis (4-cyanovaleric acid) (ACVA, Sigma, Metairie, LA, USA, 98%), 2,2′-Dithiodipyridine (Aladdin, Shanghai, China, 98%), 2-Mercaptoethanol (Sigma, Metairie, LA, USA, 99%). *N*′-dicyclohexylcarbodiimide (DCC, Sigma, Metairie, LA, USA, 99%), 4-(dimethylamino)pyridine (DMAP, Sigma, LA, USA, 99%), Tris(2,2-bipyridine) dichlororuthenium(II) hexahydrate (Aladdin, Shanghai, China, 98%), *N*-isopropylacrylamide (NIPAAm, Sigma, LA, USA, 98%) were recrystallized twice in hexane and toluene prior to use. 2-ethyl-1-hexanol (Sigma, LA, USA, ≥98%), ALP and 4-nitrophenyl phosphate (pNPP) (Sigma, Metairie, LA, USA), PEG-bis (*N*-succinimidyl succinate) (Sigma, Metairie, LA, USA), Chloroauricacid (HAuCl_4_·H_2_O, Energy Chemical, Shanghai, China, 98%), sodiumhydroxide (NaOH, Sigma, Metairie, LA, USA) were purchased from Guangfu Technology Development Co. Ltd., Tianjin, China). Albumin from bovine serum (BSA, isoelectric point = 4.6) (Sigma, Metairie, LA, USA, ≥98%) were used as received without further purification.

### 2.2. Synthesis of BSA-AuNCs

BSA (250 mg) was dissolved in deionized water (5 mL) under stirring. Chloroauric acid (1.25 mmol) was added to the solution drop wise; after 30 min incubation in 37 °C, the pH of this mixture was adjusted to 12 by adding NaOH (0.125 mol) and the bottle was incubated in 37 °C oven for 12 h. Then, the solution was dialyzed (dialysis tubing 8–14 kDa MWCO) extensively against Milli-Q water.

### 2.3. Synthesis of PNIPAAm by RAFT Polymerization

Mercaptopyridine-activated trithiol-RAFT agent (3 mg, 6.7 µmol), tris (2,2′-bipyridine) dichlororuthenium(II) hexahydrate (0.1 mg, 0.13 µmol), NIPAAm (900 mg, 7.97 mmol) and acetonitrile (2 mL) were added to a 10 mL of round-bottom flask. The flask was then sealed, and the solution was degassed via four freeze pump-thaw cycles. The polymerization was carried out at UV-irradiation (395 nm) for 36 h and purified by three times precipitation in diethylether/hexane (2:1 volume ratio). The obtained polymer was characterized by ^1^H-NMR spectroscopy in CDCl_3_ (Mn ≈ 30,000 g/mol).

### 2.4. Synthesis of BSA-Au NCs-PNIPAAm Nanoconjugates

PNIPAAm was added to a stirred solution of BSA-Au NCs. The mixed solution was stirred for 0.5 h, and then purified by using a centrifugal filter (MWCO 50 kDa) to remove any unreacted PNIPAAm and salts. After freeze-drying, the BSA-Au-PNIPAAm conjugates were obtained.

### 2.5. Preparation of Hybrid Microcapsules

The microcapsules were prepared by mixing an aqueous BSA-Au NCs-PNIPAAm solution with 2-ethyl-1-hexanol followed by shaking the mixture by hand for 10 s. The samples were prepared at a constant aqueous/oil volume fraction of 0.06. 0.06 mL of aqueous BSA-Au NCs-PNIPAAm (pH 8.5, sodium carbonate buffer) was mixed with 1.0 mL of the oil.

### 2.6. Transferring Hybrid Microcapsules into Aqueous Solution

The hybrid microcapsules were then cross-linked in the continuous oil phase by addition of PEG-bis(*N*-succinimidyl succinate) (0.5 mg), which reacted with free remanent primary amine groups of BSA. The transfer of the cross-linked microcapsules into water was achieved as follows. After 3 h sedimentation, the upper clear oil layer was discarded, and 1 mL of 70% ethanol was added. The hybrid microcapsules were washed three times by 70% ethanol via centrifugation-disperse process, then washed by Milli-Q water to complete the phase transfer process.

### 2.7. Calculating the Membrane Permeability of Hybrid Microcapsules and Percentage Diffusion

The experiments were performed by mixing 10 µL of 0.2 mg/mL FITC-dextran solution with 20 µL of hybrid microcapsules aqueous dispersion, 20 µL deionized water and incubating the mixture at 25 °C for 30 min. The uptake or exclusion of the FITC-dextran macromolecules was directly observed by a combination of fluorescence microscopy images. The image pairs are for hybrid microcapsules incubated at 25 °C in the presence of FITC-dextran with a molecular weight of 4, 10, 20, 40, 70, 150, 500, or 2000 kDa, respectively).

### 2.8. Calculating the Membrane Permeability of Hybrid Microcapsules under Redox-Environment and Percentage Diffusion

The experiments were performed by mixing 10 µL of 0.2 mg/mL FITC-dextran solution with 20 µL of hybrid microcapsules aqueous dispersion, 5 µL H_2_O_2_ aqueous dispersion, 15 µL deionized water and incubating the mixture at 25 °C for 30 min. The uptake or exclusion of the FITC-dextran macromolecules was directly observed by a combination of fluorescence microscopy images. The image pairs are for hybrid microcapsules incubated at 25 °C in the presence of FITC-dextran with a molecular weight of 4, 10, 20, 40, 70, 150, 500, or 2000 kDa, respectively).

### 2.9. The Catalytic Activity of Hybrid Microcapsules

The substrate disodium 4-nitrophenyl phosphate (pNPP) was employed to determine the catalytic ability of the encapsulated ALP by monitoring the product p-nitrophenol at 405 nm in the UV-vis spectra. Then, 6 µL ALP (10 mg/mL) was encapsuled in hybrid microcapsules, the solution was attenuated to 1mL, and then 400 µL pNPP(5mg/mL) was added to solution, kept at 25 °C for 30 min. Mnitoring the product at 405 nm in the UV-vis spectra.

Under redox-environment, 6µL ALP (10 mg/mL) was encapsuled in hybrid microcapsules; the solution was attenuated to 950 µL and 50 µL (1 × 10^−4^ M and 1 × 10^−2^ M) H_2_O_2_ was added to the solution, kept at 25 °C for 60 min. Then, 400 µL pNPP (5 mg/mL) was added to above solution, kept at 25 °C for 30 min. The product was monitored at 405 nm in the UV-vis spectra.

### 2.10. Characterizations

^1^H-NMR spectra were recorded on Bruker Advance-400 MHz (Lausanne, Switzerland), Transmission electron microscopy (TEM) analysis was undertaken on a JEM-1400 (Akishima-shi, Japan). SEM images were obtained on a HITACHI UHR FE-SEMSU8000 (Shimadzu, Japan) and UV-Vis spectra were measured on a PerkinElmer spectrophotometer (Lambda 750S, MA, USA).

## 3. Results and Discussion

**Figure 1:**
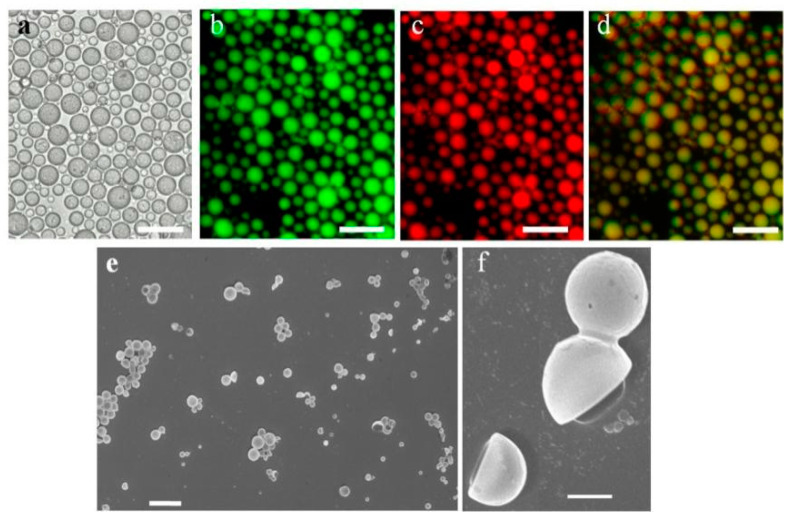
(**a**) Optical microscope image of polymeric microcapsules (HPs) in oil phase, (**b**–**d**) fluorescence microscope images of HPs in water-in-oil solution, showing (**b**) green and (**c**) red fluorescence derived from encapsulating fluorescein isothiocyanate labeled dextran (FITC-Dextran, MW 500 kDa) and Au NCs, respectively; (**d**) the overlapping image of (**b**,**c**); and SEM showing a hollow (**e**) and stabilized structure (**f**) of HPs. Scale bars in (**a**–**d**) are 50 μm, (**e**,**f**) are 20 μm and 2 μm, respectively.

First, HPs were prepared by interfacial assembly of BSA-Au NCs-PNIPAAm conjugates at the water droplet/2-ethyl-1-hexanol (oil) interface according to our previously reported method (Figure S1, Supporting Information) [10,11,12,13,14,15]. The hollow HPs with the size ranging from 9 μm to 23 μm were generated throughout the whole process by using the BSA-Au NCs-PNIPAAM (Figure 1a). Also, by loading a fluorescence isothiocyanatelabeled dextran (FITC-Dextran, MW 500 kDa) inside the HPs, the corresponding fluorescence microscopy images suggested that the formed HPs were in the form of water-in-oil emulsion (Figure 1b). For the constructed HPs, given the Au clusters in the membrane of HPs, this will endow the generated HPs with photoluminescence behavior (Figure 1c,d).

**Figure 2:**
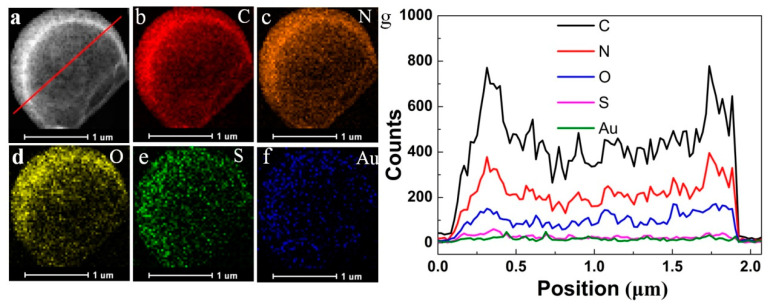
High-resolution transmission electron microscopy (HRTEM) showing continuous and robust HPs membrane (**a**). (**b**–**f**) The C, N, O, S, and Au elemental distribution. (**g**) EDS line profile analysis of C, N, O, S and Au elemental distribution in (**a**). Scale bars in (**a**–**f**) are 1 μm, respectively.

Moreover, the generated HPs were also confirmed by scanning electron microscopy (SEM) (Figure 1e,f), which clearly demonstrated the formed hollow structure and high stability under the environment of vacuum. The high-resolution transmission electron microscopy (HRTEM) image (Figure 2a) indicated clearly that the shell of HPs consisted of a flexible ultrathin membrane that was structurally robust even when dried under vacuum. Meanwhile, proteinosomes, polymersomes, and liposomes (Table S1) could not keep its stereochemical structure under vacuum, also indicating that the generated HPs showed great potential in the field of preserve in severe conditions. Also, the corresponding elemental mapping analysis and line profile in the HRTEM image (Figure 2 b–g) also demonstrated the homogeneous distribution of C, N, O and S, as well as the homogeneous distribution of Au (the characteristic elements in gold clusters) in the membrane of the HPs.

**Figure 3:**
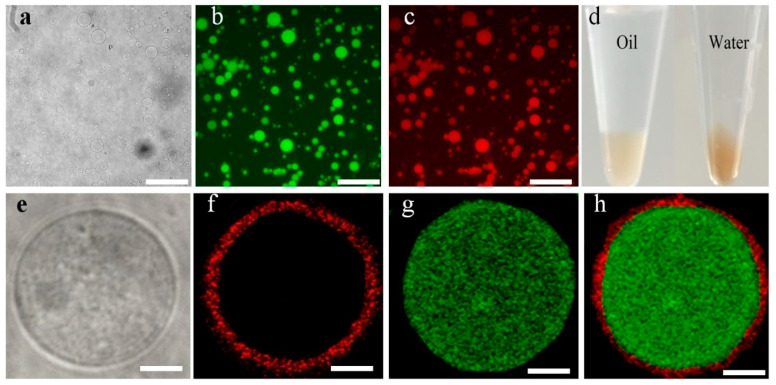
Optical microscope image of original HPs in water phase (**a**), fluorescence microscope images of HPs in water/oil solution, showing green (**b**) and red fluorescence (**c**) derived from encapsulating fluoresce in isothiocyanate labeled dextran (FITC-Dextran MW 500 kDa) and Au NCs, respectively, and the HPs in oil and aqueous phase (**d**). (**e**) Confocal microscopy images showing an HP incorporating Au NCs (red, **f**), FITC-dextran (MW 500 kDa, green, (**g**) and the overlapping image (**h**) of (**f**,**g**) in the shell and core of the HP. Scale bars in (**a**–**c**) are 100 μm, (**a**–**d**) are 5 μm, respectively.

The HPs were transferred from water-in-oil to water-in-water by adding PEGbis (*N*-succinimidylsuccinate), which could react with the free primary amine groups in the conjugates. From Figure 3a, the maximum diameter of the prepared HPs can reach 25 μm, which makes the HPs very stable. From Figure 3b,c, the HPs keep the structure integrity by the fluorescence images FITC-Dextran (MW 500 kDa) encapsuled to HPs and existence of the Au NCs. After HPs were transferred from water-in-oil to water-in-water, the volume of HPs keeps the same. It is clearly that the transfer efficiency is very high (Figure 3d). The co-location of the Au NCs and FITC-dextran (MW 500 kDa) on the surface or inner part was confirmed by confocal fluorescence microscopy images (Figure 3e). The confocal fluorescence images indicated that Au NCs, part of building block, were on the surface of HPs (Figure 3f). Furthermore, FITC-dextran was encapsuled into the HPs (Figure 3g). These HPs could show two different emission and existence of their locations at the same time (Figure 3h).

We used different molecular weights of FITC-dextran ranging from 4 to 2000 kDa to assess the permeability of the generated microcapsules. By mixing 0.1 mg/mL of different FITC-dextran with the measured microcapsules and incubating for 30 min, the fluorescence microscopy images were captured from which the fluorescence intensity difference between inside and outside microcapsule was measured by the ImageJ software. The experiments were performed by mixing 5 µL of 0.1 mg/mL FITC-dextran solution with 50 µL of the HPs aqueous dispersion. After incubating the mixture at room temperature for 30 min, the corresponding fluorescence microscopy images were captured under the same condition (a–h) in the presence of FITC-dextran with a molecular weight of 4, 10, 20, 40, 70, 150, 500, or 2000 kDa, respectively (Figure S2, Supporting Information). The corresponding fluorescence intensity line profiles of selected microcapsules are shown under each fluorescence image.

**Figure 4:**
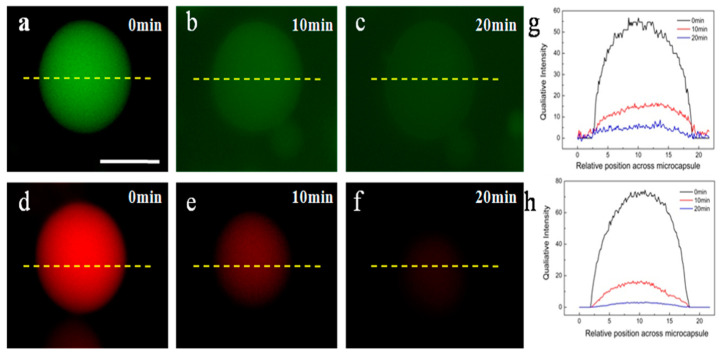
(**a**–**f**) Fluorescence microscopy image of HPs at different time in the presence of 1 × 10^−2^ M H_2_O_2_. (**g**,**h**) Corresponding intensity profiles for single HPs shown in (**a**–**c**,**d**–**f**). Scale bar is 10 μm.

The experiments were performed by mixing H_2_O_2_ with the HPs aqueous for 30 min. The corresponding fluorescence intensity lines profiles of selected microcapsule are shown under each fluorescence image are obtained (Figure S3, Supporting Information). Compared with Figures S2 and S3, the results indicated that the original constructed HPs only allowed the compound with the molecular weight of less than 20 kDa diffuse inside, while after adding H_2_O_2_, as anticipated, the molecular weight cutoff of the membrane increased to be 55 kDa (Figure S4, Supporting Information) which then could allow the corresponding encapsulated FITC-Dextrane. This phenomenon is mainly seen by removing Au NCs and PNIPAAm from the membranes, the corresponding “coating” could be disappeared. In this work, gold-sulfide bond (Au-S) is not stable under redox-environment, which could be broken by adding hydrogen peroxide (H_2_O_2_). Consequently, a fast and huge permeability modulation of the microcapsules was realized. Therefore, Au-S bounds could be broken by adding H_2_O_2_ into the system, the removal of the Au NCs and PNIPAAm from the membrane could result in a further enhancement of the permeability. To confirm so, the removal of the AuNCs and PNIPAAm from the membrane was also monitored by the fluorescence microscopy images (Figure 4). From Figure 4, the fading of the green and red fluorescence in the membrane domain indicated the cleavage of the conjugated PNIPAAm and Au NCs. Obviously, such an on-demand modulation of the membrane permeability would easily be used as an “adjuster” when doing enzyme catalysis as a microreactor. 

In this work, phosphatase (ALP) was encapsuled into HPs and p-nitrophenyl phosphate (pNPP) as substrate, which was employed to determine the catalytic ability by monitoring the yellow product p-nitrophenol at 405 nm. In comparison with original HPs, the catalytic efficiency was increase 4.06- and 2.22-fold after adding 1 × 10^−2^ M and 1 × 10^−4^ M H_2_O_2,_ respectively (Figure S5, Supporting Information). Due to the Au NCs and PNIPAAm being removed from the membrane, the substrate easily enters the microcapsules and could easily react with enzyme. This helps us to design some special reactions in the microcapsules (micro-factory) to increase/decrease efficiency by changing the permeability of membrane.

## 4. Conclusions

In summary, a BSA-AuNCs-PNIPAAm building block was used to generate hybrid hollow polymeric microcapsules by Pickering emulsion, with sizes ranging from 9 μm to 23 μm. The generated HPs showed a clear stimuli responsive behavior against redox species, contributed by the disulfide bond and Au-S linkage between BSA-PNIPAAm and AuNCs-PNIPAAm in the membrane. Significantly, via an obviously triggered stimuli, a modulation of the membrane permeability was realized as shown in the adjustable release rate of the encapsulated FITC-dextran. Also, the permeability of the HPs was able to be turned up from 20 kDa to 55 kDa without or with H_2_O_2_. Moreover, the catalytic reaction rate of the ALP loaded bared HPs by using pNPP as substrates was increased with different concentrations of H_2_O_2_. In view of the significant role of membrane permeability in the fields of microreactor and artificial cell, this novel system is expected to enrich the design of pharmaceutical specific synthesis, production separation, and protocell models. Mover, it can modulate the reaction of diffusing across the boundary membrane to realize cell communication or information transfer.

## Data Availability

The data presented in this study are available on request from the corresponding author.

## Supplementary Materials

The following are available online, Figure S1. Assembly of BSA-Au NCs-PNIPAAm and hybrid microcapsules(a), UV-vis absorption (b) and emission (c) spectra of the as prepared BSA-Au NCs, HRTEM images of BSA-Au NCs (d), the enlarge image of Au NCs (e). The scale bars in d and e are 20 and 5 nm; Table S1 Comparison of morphology under vacuum; Figure S2. Permeability of the HPs based on the diffusion of the fluorescent-labeled dextrans (FITC-dextran) with molecular weights range from 4 to 2000 kDa; Figure S3. Permeability of the HPs under redox environment based on the diffusion of the fluorescent-labeled dextrans (FITC-dextran) with molecular weights from 4 to 2000 kDa; Figure S4. Plot of percentage diffusion after 30 min for the HPs-Au incubated in the presence of FITC-dextran of different molecular weight with (red bars) or without (black bars) H_2_O_2_ respectively; Figure S5. The catalytic reaction rate of the ALP loaded bared HPs-Au, with 1 × 10^−4^ M and 1 × 10^−2^ M H_2_O_2_ by using pNPP as substrates, respectively.

## Sample Availability

Samples of the compounds are not available from the authors.
