# Supplementary material for: Surface Permeability of Membrane and Catalytic Performance Based on Redox-Responsive of Hybrid Hollow Polymeric Microcapsules
Source: Molecules. 2021 Jan 26;26(3):633. doi: 10.3390/molecules26030633 (PMC7866142; doi:10.3390/molecules26030633)
Supplement: Supplementary file 1 [file molecules-26-00633-s001.pdf]

Supporting information

Surface permeability of membrane and catalytic performance based on redox-responsive of hybrid hollow polymeric microcapsules

Guangyu Wu[1, 2, 3, 4, 5]*, Jingyi Wang[1], Qi Liu[1], Ran Lu[1], Yuhan Wei[1], Feng Cheng[6], Jiangang Han[1, 5]*, Weinan Xing[1, 5]* and Yudong Huang[6]

1 College of Biology and the Environment, Co-Innovation Center for the Sustainable Forestry in Southern China, Nanjing Forestry University, Nanjing 210037, China

2 Key Laboratory of Functional Polymer Materials, Ministry of Education, Nankai University, Tianjin 300071, China

3 State Key Lab of Fine Chemicals, Dalian University of Technology, Dalian 116024, China

4 Jiangsu Provincial Key Laboratory of Palygorskite Science and Applied Technology, Huaiyin Institute of Technology, Huaian 223003, China

5 National Positioning Observation Station of Hung-tse Lake Wetland Ecosystem in Jiangsu Province, Hongze, Jiangsu 223100, China

6 MIIT Key Laboratory of Critical Materials Technology for New Energy Conversion and Storage, State Key Laboratory of Urban Water Resource and Environment, School of Chemistry and Chemical Engineering, Harbin Institute of Technology, Harbin 150001 ,China

* E-mail: gywuchem@163.com; xingwn@njfu.edu.cn; hjg@njfu.edu.cn

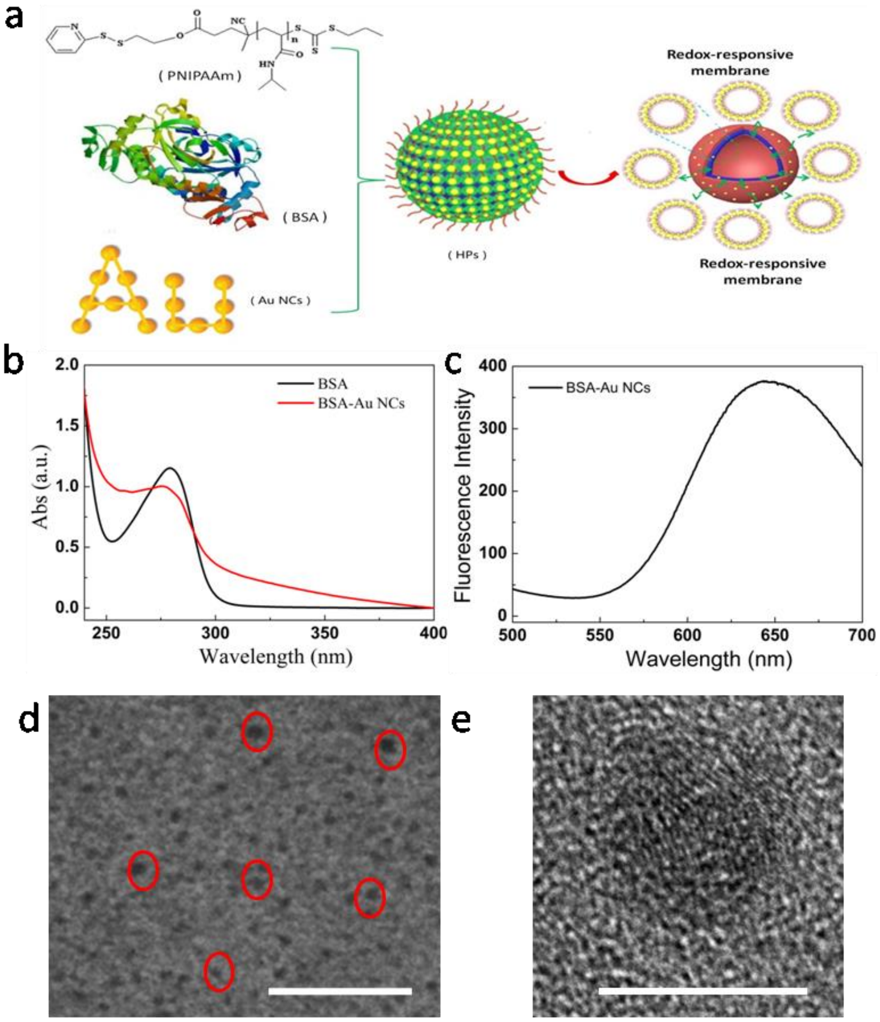

Figure S1. Assembly of BSA-Au NCs-PNIPAAm and hybrid microcapsules(a), UV-vis absorption (b) and emission (c) spectra of the as prepared BSA-Au NCs, HRTEM images of BSA-Au NCs (d), the enlarge image of Au NCs (e). The scale bars in d and e are 20 and 5 nm.

Table S1 Comparison of morphology under vacuum

Sample	Size	Morphology	Reference
Proteinsomes	20-50 µm	Ultrathin membrane	1
Polymersomes	900nm	Collapsed vesicles	2
Liposomes	91nm	Collapsed vesicles	3
Hybrid Microcapsules	9-23 µm	3D Hollow Microcapsules	This Work

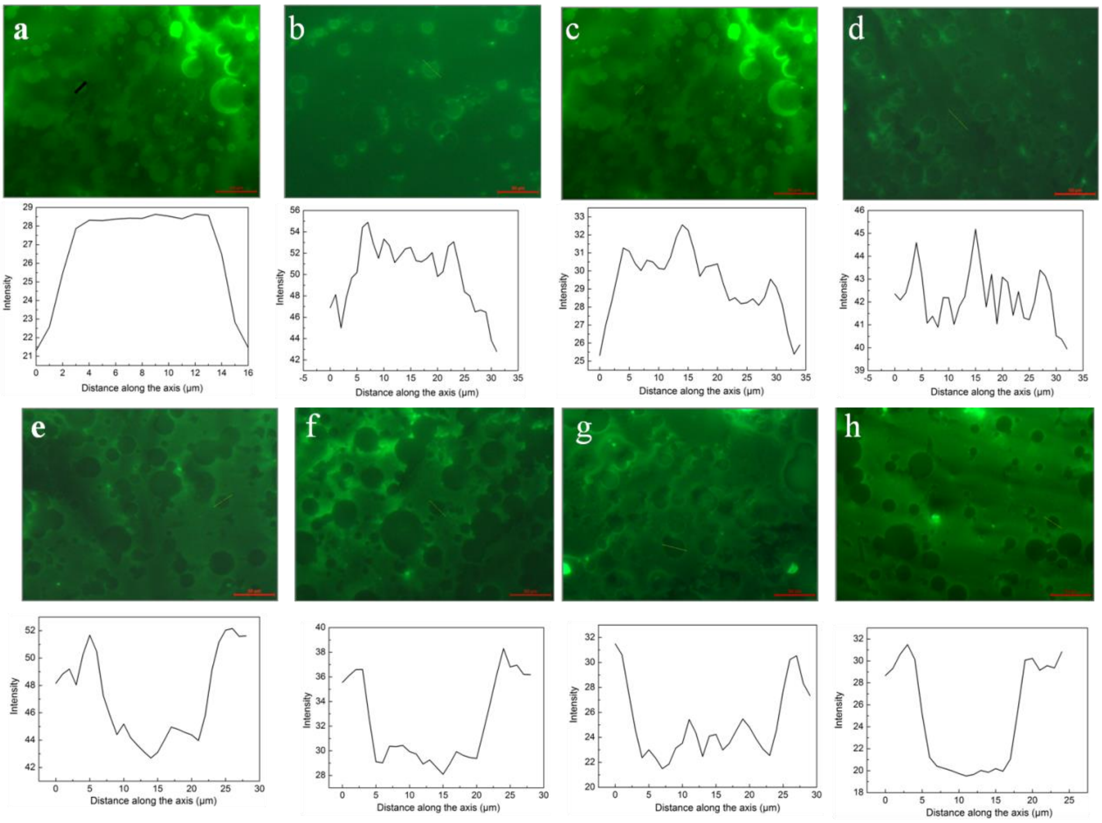

Figure S2. Permeability of the HPs based on the diffusion of the fluorescent-labeled dextrans (FITC-dextran) with molecular weights range from 4 to 2000 kDa.

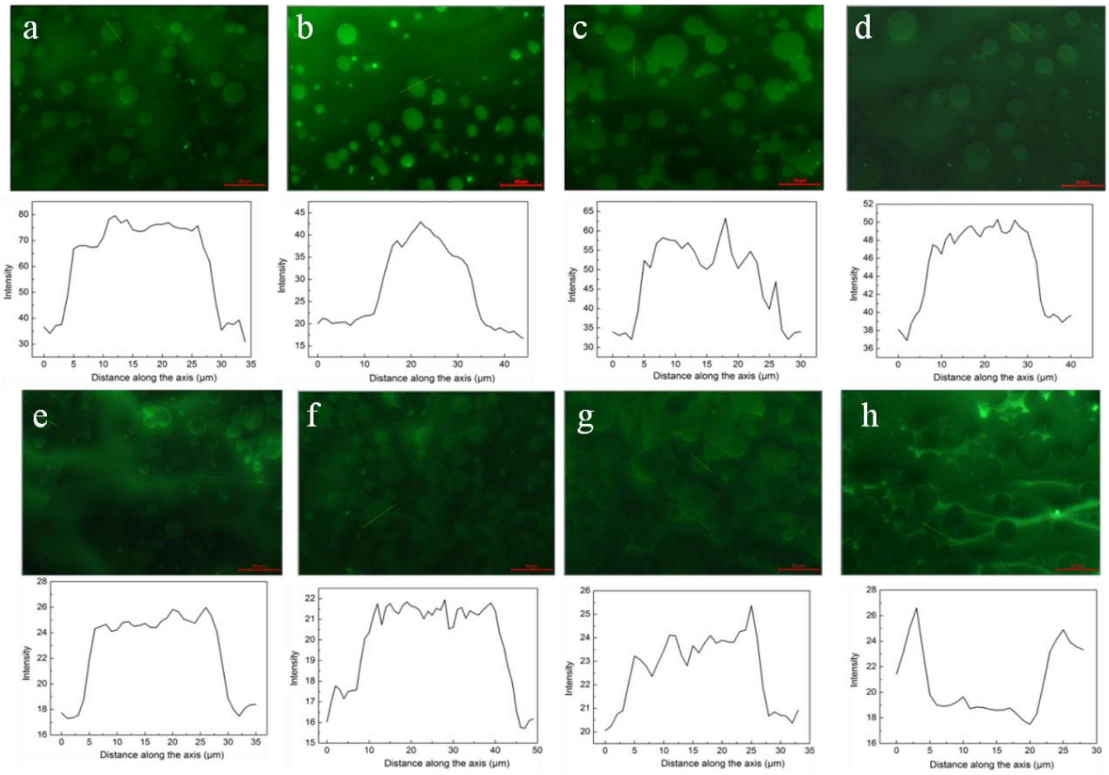

Figure S3. Permeability of the HPs under redox environment based on the diffusion of the fluorescent-labeled dextrans (FITC-dextran) with molecular weights from 4 to 2000 kDa.

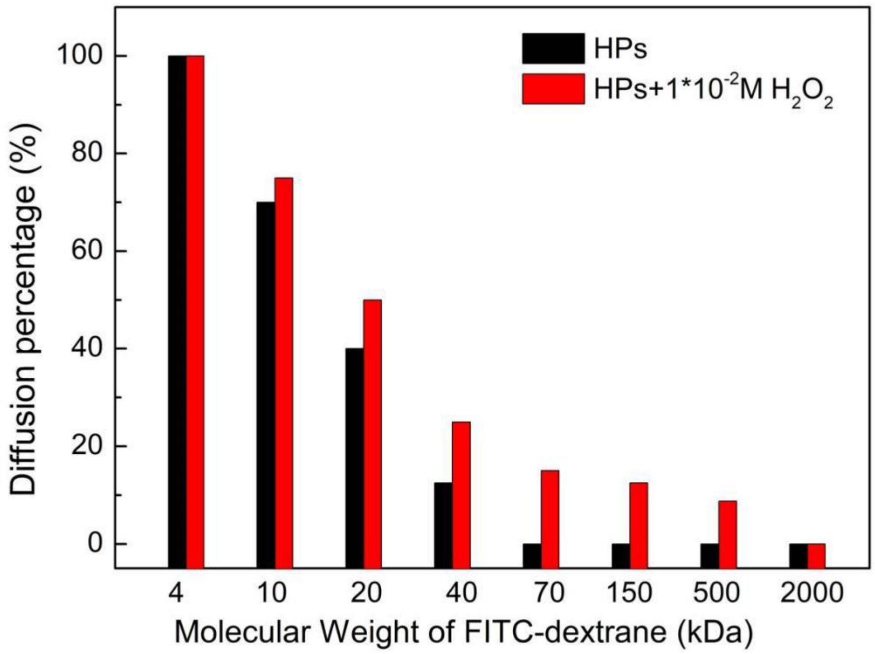

Figure S4. Plot of percentage diffusion after 30 min for the HPs-Au incubated in the presence of FITC-dextran of different molecular weight with (red bars) or without (black bars) H_2O_2 respectively.

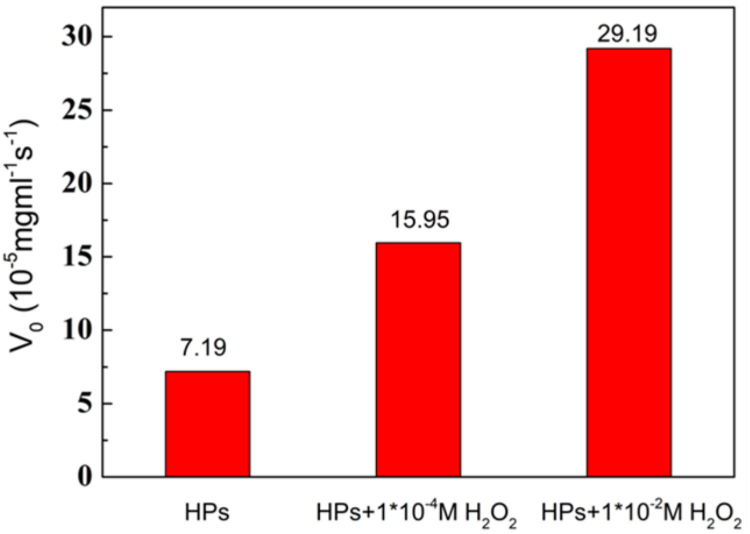

Figure S5. The catalytic reaction rate of the ALP loaded bared HPs-Au, with 1*10^{-4}M H_2O_2 and 1*10^{-2}M by using pNPP as substrates, respectively

References:

[1] Zhou, P; Liu, XM ; Wu, GY; Wen, P ; Wang, L; Huang, YD; Huang, X. Programmable Modulation of Membrane Permeability of Proteinosome upon Multiple Stimuli Responses. ACS Macro Letters, 2016, 5(8)：961-966.
[2] Cao, C ; Chen, F; Garvey, CJ ; Stenzel, MH. Drug-Directed Morphology Changes in Polymerization-Induced Self-Assembly (PISA) Influence the Biological Behavior of Nanoparticles. ACS Applied Materials & Interfaces, 2020, 12(27): 30221-30233.
[3] Carone, M; ; Cangiotti, M; Ottaviani, MF ; Carloni, R ; Appelhans, D. Langmuir, 2020, 36(43): 12816-12829.
